# Morin Protects Channel Catfish From *Aeromonas hydrophila* Infection by Blocking Aerolysin Activity

**DOI:** 10.3389/fmicb.2018.02828

**Published:** 2018-11-21

**Authors:** Jing Dong, Yongtao Liu, Ning Xu, Qiuhong Yang, Xiaohui Ai

**Affiliations:** ^1^Yangtze River Fisheries Research Institute, Chinese Academy of Fishery Sciences, Wuhan, China; ^2^Key Laboratory of Control of Quality and Safety for Aquatic Products, Ministry of Agriculture, Beijing, China

**Keywords:** *Aeromonas hydrophila*, aerolysin, morin, anti-virulence, antibiotics

## Abstract

*Aeromonas hydrophila* (*A. hydrophila*) is an opportunistic bacterial pathogen widely distributed in the environments, particular aquatic environment. The pathogen can cause a range of infections in both human and animals including fishes. However, the application of antibiotics in treatment of *A. hydrophila* infections leads to the emergence of resistant strains. Consequently, new approaches need to be developed in fighting this pathogen. Aerolysin, the chief virulence factor produced by pathogenic *A. hydrophila* strains has been employed as target identifying new drugs. In our present study, we found that morin, a flavonoid without anti-bacterial activity isolated from traditional Chinese medicine, could directly inhibit the hemolytic activity of aerolysin. To determine the binding sites and the action of mechanism of morin against AerA, several assays were performed. Ser36, Pro347, and Arg356 were identified as the main binding sites affecting the conformation of AerA and resulted in block of the heptameric formation. Moreover, morin could protect Vero cells from cell injury mediated by aerolysin. *In vivo* study showed that morin could provide a protection to channel catfish against *A. hydrophila* infection. These results demonstrated that morin could be developed as a promising candidate for the treatment of *A. hydrophila* infections by decreasing the pathogenesis of *A. hydrophila*.

## Introduction

*Aeromonas hydrophila* (*A. hydrophila*) is a gram-negative aquatic bacterium widely distributed in aquatic water worldwide leading to a number of diseases in fish (Grim et al., 2014). Outbreak of *A. hydrophila* infections resulted in high mortality and severe economic losses to the aquaculture industry all over the world (Zhang et al., 2018). Moreover, the pathogen can transmit from diseased fish, contaminated water or uncooked food to human (Rama Devi et al., 2016). Although *A. hydrophila* is not a typical pathogen for human, it has been reported to be responsible for a range of infections including septicemia, wound infections, burn-associated sepsis, and respiratory tract infections (Santos et al., 1999; Casabianca et al., 2015). Antibiotics are the main approach in the treatment of infections caused by bacterium (Dong et al., 2017a). However, the abuse of antibiotics in aquaculture leads to emergence of antibiotic resistance and environmental pollution (Zhang et al., 2015; Stratev and Odeyemi, 2016). The spread of antibiotic resistance can reduce the effect of antibiotics in the treatment of infections caused *A. hydrophila* and is a potential threaten to human health (Stratev and Odeyemi, 2016). Therefore, there is an urgent need for new approaches against *A. hydrophila* infections.

As is known, pathogenic *A. hydrophila* can produce a number of virulence factors including proteases, hemolysin (HlyA), aerolysin (AerA), enterotoxins, and acetylcholinesterase which contribute to the pathogenicity of the bacterium (Bi et al., 2007). AerA, a pore forming toxin with hemolytic, cytotoxic and enterotoxic activities produced by all pathogenic *A. hydrophila* strains, plays a critical role in the pathogenicity of *A. hydrophila* and has been identified as a marker of pathogenic *A. hydrophila* strains (Howard and Buckley, 1982; Abrami et al., 2003). The toxin was secreted as a 52 kDa precursor without activity named proaerolysin (pAerA), then the toxin can be activated after cleaving a flexible 43-residue loop near the C-terminus by trypsin or furin (Abrami and van Der Goot, 1999; Iacovache et al., 2011). After binding on the glycosylphosphatidylinositol (GPI) anchor of target cells, the toxin is concentrated and promote forming oligomerization with channel pore that can insert into the membrane (Iacovache et al., 2006). AerA is one of the most well-known and characterized pore forming toxins, previous studies have shown that AerA is the main virulence factor of *A. hydrophila* (Wu and Guo, 2010).

**FIGURE 1:**
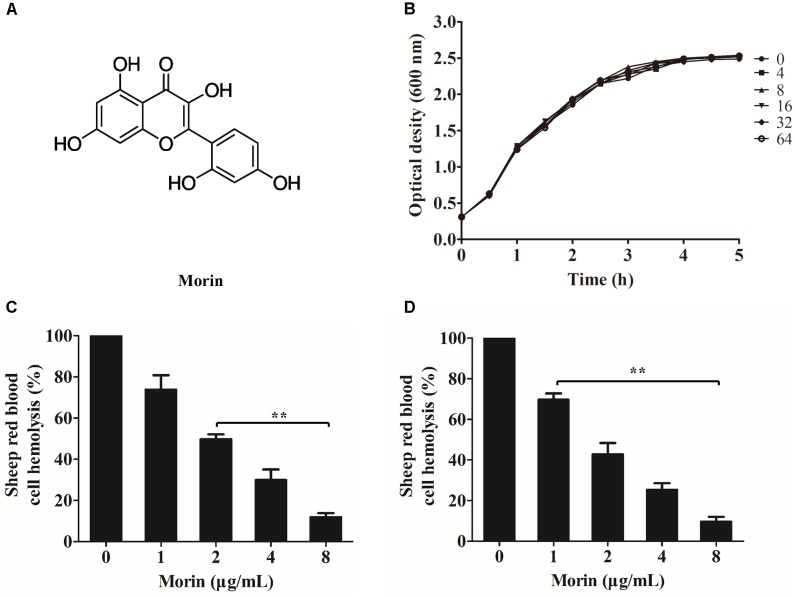
Morin inhibits the hemolytic activity induced by AerA. **(A)** Chemical structure of morin. **(B)** Growth curves of *A. hydrophila* strain XS-91-4-1 co-cultured with different concentrations of morin. **(C)** Inhibition of hemolytic activity of *A. hydrophila* supernatants co-cultured with morin. **(D)** The inhibitory effect of morin against purified AerA. Data shown in panels **(C,D)** were presented as mean value ± SD of three independent assays. ^∗^*p* < 0.05 and ^∗∗^*p* < 0.01 when compared with the drug-free supernatants or AerA.

Morin (Figure 1A), a Flavonoid can be isolated from several traditional Chinese medicine, has a series of biological activities such as antioxidant, anti-apoptotic, and anti-inflammatory activity (Huang et al., 2014). In this paper, we found that morin, without anti-*A. hydrophila* activity, could inhibit the hemolytic activity by hindering the heptameric formation of AerA. Then the binding sites were calculated by molecular dynamics simulations and validated by fluorescence quenching assay. Moreover, we demonstrated that morin could protect Vero cells from AerA mediated injury and decrease the mortality of channel catfish (*Ictalurus punctatus*) infected with *A. hydrophila*.

## Materials and Methods

### Micro-Organism and Reagents

*Aeromonas hydrophila* strain XS-91-4-1 was isolated from diseased *Hypophthalmichthys molitrix* XS-91-4-1 was cultured in brain heart infusion (BHI) medium at 28°C for experiments described below. Morin (purity > 98%) and enrofloxacin (purity > 98%) were obtained from National Institutes for Food and Drug Control (Beijing, China). Stock solutions of both drugs were prepared in dimethyl sulfoxide (DMSO, Sigma-Aldrich, St. Louis, MO, United States). For *in vivo* study, morin was dissolved in sterile PBS. The minimum inhibitory concentrations (MIC) of morin and enrofloxacin were determined by broth micro-dilution method recommended by Clinical and Laboratory Standards Institute [CLSI] (2009).

### Growth Curves

*Aeromonas hydrophila* strain XS-91-4-1 was cultured in BHI medium at 28°C to obtain optical density at 600 nm (OD_600__nm_) of 0.3, and then the culture was aliquoted into a 100-ml flask and a series of morin with a final concentration of 0, 4, 8, 16, 32, and 64 μg/mL were added. The mixture was further cultured for 5 h at 28°C. Cell growth was evaluated by recording the OD_600__nm_ values at intervals of 30 min using a spectrophotometer.

### Hemolytic Assays

Hemolytic activities were performed using purified AerA and supernatants of *A. hydrophila* co-cultured with different concentrations of morin ranging from 1 to 8 μg/mL. Hemolytic assays were performed according to previous reports (Dong et al., 2013; Dong et al., 2017a). For hemolytic assay of co-cultured supernatant, 100 μL of trypsin treated supernatants were combined with 25 μL defibrinated sheep red blood cells (5 × 10^6^ cells/ml) in hemolytic buffer (20 mM Tris, 150 mM NaCl, pH 7.2). For hemolytic activity of purified AerA, 2 μL of concentrated protein were added into 975 μL hemolytic buffer, 25 μL defibrinated sheep red blood cells were added after incubation at 37°C for 15 min. Following centrifugation, the hemolytic activities of the mixtures were determined by measuring the absorption at 543 nm after further incubated at 37°C for 20 min. Morin-free group was served as 100% hemolytic control.

### Western Blot Analysis

Western blot assay was performed as reported elsewhere (Dong et al., 2013). In brief, *A. hydrophila* strain XS-91-4-1 was incubated at 28°C in BHI medium with morin at concentrations of 1, 2, 4, and 8 μg/mL to OD_600__nm_ = 1.5. Supernatants of the culture were centrifuged and the supernatants were used for sodium dodecyl sulfate (SDS)-polyacrylamide (12%) gel electrophoresis, BHI medium was employed as negative control. Then a semi-dry transfer cell was used to transfer the proteins onto a polyvinylidene fluoride membrane. After blocking the membrane with 3% skim milk for 60 min at room temperature, an anti-aerolysin primary polyclonal antibody was incubated with the membrane at 1:1000 dilution overnight at 4°C, and followed incubating with a HRP-conjugated secondary goat anti-rabbit antiserum (diluted 1:5000) for 1 h. The blots were then treated with ECL western blotting detection regents.

### Molecular Docking and Molecular Dynamics

The binding mode between the morin and the pAerA was analyzed by molecular docking method using AutoDock Vina 1.1.2 (Trott and Olson, 2010). The crystal structure of the pAerA (PDB ID: 1PRE) was obtained from Protein Data Bank^1^. The structure of morin was drawn by ChemBioDraw Ultra 12.0 and ChemBio3D Ultra 12.0 software. The files for docking were generated by the AutoDockTools 1.5.6 package (Sanner, 1999; Morris et al., 2009). Structures of morin were prepared for docking by merging non-polar hydrogen atoms and defining rotatable bonds. The search grid of the pAerA was identified as center_x: 9.973, center_y: 49.913, and center_z: 27.774 with dimensions size_x: 47.25, size_y: 47.25, and size_z: 47.25. The exhaustive value was set to 20 to improve the accuracy of the docking. Then the docking results were modified by performance of an MD study.

MD simulations of docked pose were performed by the Amber 14 and AmberTools 15 programs on Dell Precision T5500 workstation as described previously (Gotz et al., 2012; Pierce et al., 2012; Salomon-Ferrer et al., 2013). The automatic topologies, parameters and calculation of partial charges were generated by ACPYPE, a tool based on ANTECHAMBER (Wang et al., 2004, 2006; Sousa da Silva and Vranken, 2012). Then, morin was prepared by forcefield “leaprc.gaff,” while “leaprc.ff14SB” was employed for the preparation of pAerA. The “SolvateOct” command with the minimum distance was performed to put the reaction system in a rectangular box with TIP3P water. The pAerA–morin system was first energy relaxed by 2000 steps of steepest descent and conjugate gradient energy minimization, and then the solvated complex was equilibrated by a 500 ps of heating, and 500 ps of density equilibration with weak restraints. At last, 40 ns of MD simulations were carried out.

### Mutagenesis of the pAerA Protein

Plasmid encoding wild-type (WT) pAerA was constructed as we reported previously (Dong et al., 2017b). Plasmid encoding S36A-pAerA, P347A-pAerA, and R356A-pAerA were conducted according to the instruction of the QuikChange site-directed mutagenesis kit (Stratagene, CA, United States) from WT-pAerA plasmid. Prime pairs for the mutant were listed in Supplementary Table 1. Then protein expression and purification were performed according to previous report (Dong et al., 2017b). Proteins were concentrated in storage buffer (25 mM Tris, 150 mM NaCl, pH 9.0) to avoid oligomerization by ultrafiltration. Trypsin was added in to purified proteins for activation by cleaving 43 residues at the C-terminal of the protein. After incubation at room temperature for 10 min, the reaction was stopped by addition of a 10-fold excess of trypsin/chymotrypsin inhibitor (Abrami and van Der Goot, 1999). Activated proteins were stored at -80°C for further applications.

### Binding Affinity Determination of WT-AerA and Mutants

The binding constants (*K_A_*) of morin to WT-AerA and mutants were determined by the fluorescence quenching method using a Cary Eclipse fluorescence spectrophotometer (Agilent Technologies, Santa Clara, CA, United States) as described previously (Jurasekova et al., 2009; Ibrahim et al., 2010). Briefly, A 280-nm excitation wavelength with a 5-nm band-pass and a 345-nm emission wavelength with a 10-nm band-pass were set up for the fluorescence spectrofluorimetry measurement.

### Inhibition of Oligomerization

Inhibition of oligomerization was performed as described previously. In brief, WT-AerA was mixed with morin at the same mol ratio as performed in hemolytic assays, the mixtures with a volume of 20 μL were incubated at 37°C for 15 min. Then 1 μL 1 M Hepes were added to each sample to match the pH (pH < 8) of oligomerization. The mixtures were then loaded onto 8% SDS-PAGE gels for electrophoresis after incubated at 4°C for 1 h.

### Cell Viability Assays

Vero cells were obtained from the American Tissue Culture Collection (ATCC) and were cultured in DMEM supplemented with 10% fetal bovine serum at 37°C with 5% CO_2_ in a humidified incubator. Cells with a density of 1.5 × 10^5^ per well were seeded into 96-well cell culture plates. After incubated for 16 h at 37°C with 5% CO_2_, cells were co-cultured with 100 μL of AerA at a concentration of 1 μg/mL and indicated concentrations of morin at 37°C for 24 h. All assays were performed in triplicate measurements.

The determinations of cell viability were performed by lactate dehydrogenase (LDH) release using a Cytotoxicity Detection Kit and live/dead assay with a live (green)/dead (red) regent. Dead cells were stained by propidium iodide with a fluorescent-red dye, while live cells were stained by calcein AM with a fluorescent-green dye. Cell images were taken by a confocal laser scanning microscope (Nikon, Japan). LDH activity was determined on a microplate reader (Tecan, Austria).

### Ethics Statement

All animal assays were carried out according to the experimental practices and standards developed by the Animal Welfare and Research Ethics Committee at Yangtze River Fisheries Research Institute. All the assays were approved and supervised by the animal care committee (Permit No. 20171105-009C).

### Channel Catfish Model Infected With *A. hydrophila*

Channel catfish weighing 200 ± 5 g were separated into three groups and maintained in 100 L glass aquaria tanks at 28°C for 15 days before infection. Channel catfish were infected with *A. hydrophila* by injecting 200 μL XS-91-4-1 suspension intraperitoneally. Fish injected with sterile PBS served as negative control. Infected channel catfish were administered with 25 mg/kg of morin or PBS 6 h postinfection and at 12-h intervals for 3 days. For negative control, fish were administered with PBS at the same intervals. Each group contains 10 channel catfish. The death of each group were monitored every day for 8 days.

### Statistical Analysis

The experimental data were compared by independent Student’s *t*-test with SPSS 14.0 statistical software (SPSS Inc., Chicago, IL, United States). Survival rate of channel catfish was analyzed with Kaplan-Meier test, log-rank test was carried out to analyze the significance of different groups. A *p*-value <0.05 was considered to be statistically significant.

## Results

### Morin Inhibits the Hemolytic Activity of AerA

**FIGURE 2:**
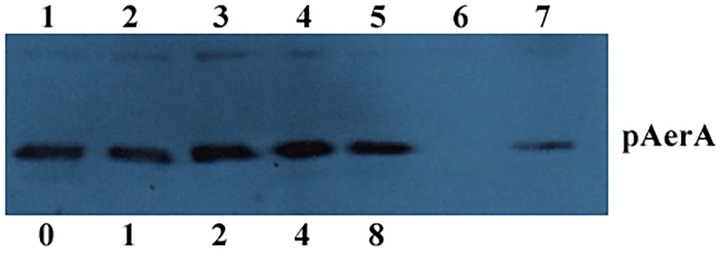
Western blot analysis of pAerA expression co-cultured with morin. Bacterial supernatants of *A. hydrophila* strain XS-91-4-1 (Lane 1–5) co-cultured with different concentrations of morin; lane 6, negative control; lane 7, purified pAerA.

Minimum inhibitory concentrations and growth curves were performed to evaluate the influence of morin on the growth of *A. hydrophila*. According to the results of MICs, morin had no evident inhibitory effect on *A. hydrophila* XS-91-4-1 strain, while the MIC of enrofloxacin was 4 μg/mL. Moreover, the results of growth curves with different concentrations of morin showed that morin could not influence the growth of *A. hydrophila* XS-91-4-1 strain from the concentration of 4–64 μg/mL (Figure 1B). In the present paper, we found that morin could not affect the expression of pAerA (Figure 2), but could inhibit the hemolytic activity of *A. hydrophila* XS-91-4-1 when co-cultured with definite concentrations of morin (Figure 1C). Before analysis, the concentrations of total protein in supernatants were determined by a BCA protein assay kit. The concentrations were 1.45, 1.42, 1.44, 1.36, and 1.47 mg/mL for the strain co-cultured with morin at concentrations of 0, 1, 2, 4, and 8 μg/mL, respectively. Furthermore, the hemolytic activity of purified AerA could be inhibited in a dose-dependent manner (Figure 1D). When treated with 8 μg/mL morin, the hemolytic activities of supernatant and purified AerA were significantly decreased to 11.86 and 9.62%, respectively. Thus, it is infer that morin can inhibit the activity of AerA directly according to these findings.

### Determination of Binding Sites by Molecular Dynamics

The AutoDock Vina 1.1.2 and Amber 14 software package were employed to analyze the potential binding mode of AerA–morin complex using molecular docking and molecular dynamics simulation methods. According to the docking results, the binding mode of AerA–morin complex was determined by 40-ns molecular dynamics simulations. As shown in Supplementary Figure 1, the root-mean-square deviation (RMSD) values of the initial structure of pAerA were calculated to confirm the dynamic stability and the rationality. The protein structures of the two systems were stabilized during the 40-ns simulation (Supplementary Figure 1).

The flexibility of residues between pAerA–morin complex and free pAerA were determined by the root mean square fluctuations (RMSF). As shown in Supplementary Figure 2, the difference of flexibilities in the binding site of pAerA was described in the absence or presence of morin. Compared with the free pAerA, a smaller degree of flexibility with a RMSF of less than 3 Å was discovered at the majority of the residues positions 91–470 in the pAerA, which suggested that the residues binding to morin became more rigid. While a big degree of flexibility with the RMSF values nearly reached to 13 Å was found at the position 2–90, which indicated that the residues became more flexible when binding to morin.

**FIGURE 3:**
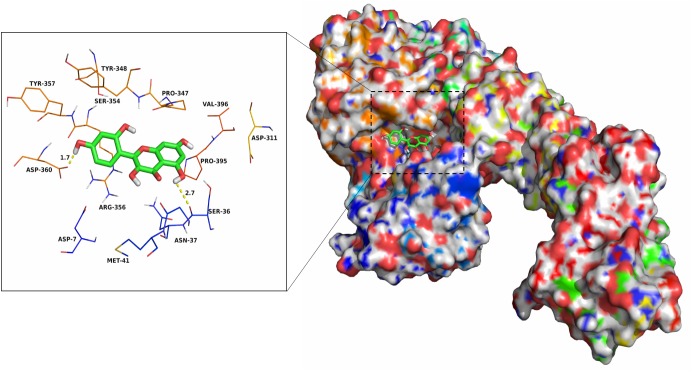
The predicted binding mode of morin in pAerA binding pocket obtained from MD simulation.

The electrostatic, *Van der Waals*, solvation, and total contribution of the residues to the binding free energy were analyzed using the MMGBSA method to evaluate the binding sites and contribution of residues to the system. The per residue interaction free energies were separated into *Van der Waals* (Δ*E_vdw_*), electrostatic (Δ*E_ele_*), solvation (Δ*E_sol_*), and total contribution (Δ*E_total_*). In the pAerA–morin complex, the residue Asp-360 have a strong electrostatic (Δ*E_ele_*) contribution, with the value of <-9.0 kcal/mol (Supplementary Figure 3). Detailed analysis showed that the residue Asp-360 is oriented to the phenyl group of the morin, and electrostatic interaction exist, leading to the anion-π interaction and one strong hydrogen bond interaction (bond length: 1.7 Å) between the pAerA and the morin (Figure 3). In addition, the residues Pro-347 and Arg-356, with the Δ*E_vdw_* of <-2.0 kcal/mol, have an appreciable *Van der Waals* interactions with the morin because of the close proximity between the residues and the morin. Except for the residues Ser-36, Ser-354, and Asp-360, *Van der Waals* interactions was found to be the major decomposed energy, apparently through hydrophobic interactions (i.e., Met-41, Pro-347, Pro-395, and Val-396). In addition, the total binding free energy for the pAerA–morin complex calculated according to the MMGBSA approach, and the an estimated binding free energy (Δ*G_bind_*) of -14.4 kcal/mol was found for morin, which revealed that morin could strongly bind to and interact with the binding site of the pAerA. Moreover, to confirm the main binding sites of morin–AerA system, Δ*G_bind_* of morin binding to active site of AerA was calculated (Table 1). In summary, the above molecular simulations explained the interactions between morin and proaerolysin, which provided useful information for identification of the pAerA inhibitors.

**Table 1:** The binding free energy (Δ*G_bind_*) based on a computational method and the values of the binding constants (*K_A_*) based on the fluorescence-quenching method.

	WT-AerA	R356A	P347A	S36A
Δ*G_bind_* (kcal/mol)	-14.4	-2.64	-2.18	-1.16
*K_A_* (1 × 10^4^) L mol^-1^	12.2	7.74	6.19	0.88
*n*	1.0998	1.0254	1.0305	0.8539

### Determination of the Mechanism of Morin Inhibiting the Activity of AerA

**FIGURE 4:**
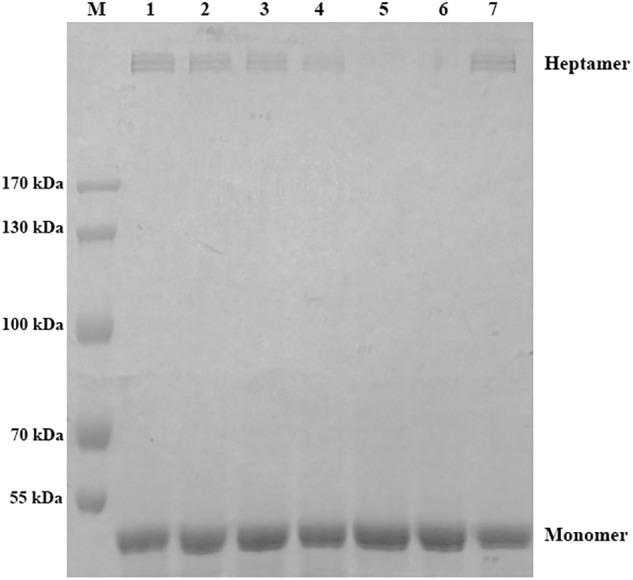
Morin prevents the formation of heptamer of AerA. AerA was treated with different concentrations of morin, oligomerization was induced by addition of Hepes. Following sodium dodecyl sulfate (SDS)-polyacrylamide gel electrophoresis (PAGE) analysis, the proteins were detected by coomassie blue staining. M, prestained protein marker; lane 1, WT-AerA; lane 2, WT-AerA plus morin at a mol ratio of 1:150; lane 3, WT-AerA plus morin at a mol ratio of 1:300; lane 4, WT-AerA plus morin at a mol ratio of 1:450; lane 5, WT-AerA plus morin at a mol ratio of 1:600; lane 6, WT-AerA plus morin at a mol ratio of 1:750; lane 7, WT-AerA.

The fluorescence quenching assay was performed to confirm the results of molecular dynamics. As shown in Table 1, Δ*G_bind_* of morin is the highest with WT-AerA, followed by Arg356A mutant, Pro347A mutant, and then Ser36A mutant, which suggesting that the ability of morin binding with WT-AerA is the strongest among all types of proteins. Similar results of the binding constants (*K_A_*) and the number of binding sites (*n*) between morin and proteins calculated by the fluorescence quenching method were obtained, indicating that findings obtained by the computational methods could be used for further research. Moreover, the oligomerization assay were carried out to analyze the mechanism of morin inhibiting the activity of AerA. As expected, morin could reduce the production of heptamer in a dose-dependent manner (Figure 4). When the mol ratio of AerA and morin reached to 1:600, no visible heptamer was observed. Moreover, the hemolytic activities of the mutants were determined, the results showed that there was no evident difference between WT-AerA and mutants (data not shown). The results indicates that mutations do not affect the formation of heptamer. Taken together, when morin binds to WT-AerA, the conformation of AerA was changed (Supplementary Figures 1, 2) and resulted in the decrease of heptamer.

### Morin Protects Vero Cells From Cell Injury Induced by AerA

**FIGURE 5:**
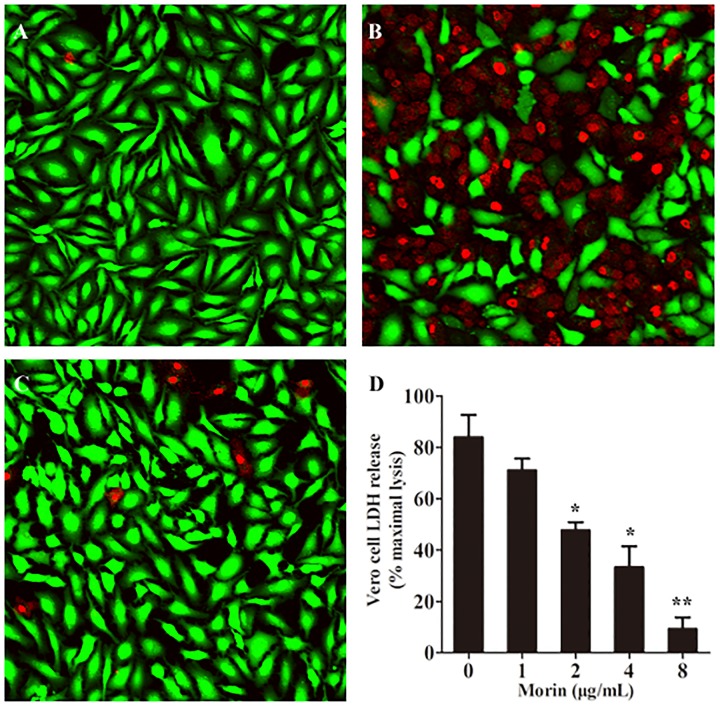
Morin protects Vero cells from AerA-induced cell injury. Vero cells were stained with live (green)/dead (red) regent and were captured by a confocal laser scanning microscope after treated with AerA with the presence or absence of morin. A fluorescent-red dye stained dead cells, while a fluorescent-green dye stained live cells. **(A)** untreated cells; **(B)** cells treated with AerA in the absence of morin; **(C)** cells treated with AerA in the presence of 8 μg/mL morin; and **(D)** LDH release by Vero cells when treated with AerA and indicated concentrations of morin. All were presented as mean value ± SD of three independent experiments. ^∗^*p* < 0.05 and ^∗∗^*p* < 0.01 when compared with the drug-free group.

It is reported that AerA can target to a number of mammalian cells, such as fibroblast like cells, lymphocytes, granulocytes, erythrocytes, and epithelial cells (Abrami et al., 2003). As reported previously, Vero cells were widely used in the measurement toxicity of aerolysin (Dong et al., 2017b). Therefore, Vero cells were employed to investigate the protective effects of morin against AerA mediated cell injury. Vero cells were stained with the live/dead regent after incubation with AerA and indicated concentrations of morin. Then cell viability was monitored by a confocal laser scanning microscope. As shown in Figure 5A, live cells were exhibited to be green, while dead cells were red (Figure 5B). When co-cultured with AerA and 8 μg/mL morin, no evident cell death was observed (Figure 5C). Moreover, the cell viability was determined by measuring the release of LDH in each sample. As expected, morin could protect Vero cells from AerA mediated cell injury in a dose dependent manner from concentrations of 1–8 μg/mL (Figure 5D). These findings demonstrated that morin could provide an *in vitro* protection to Vero cells against AerA induced cell injury.

### Morin Protects Channel Catfish From *A. hydrophila* Infection

**FIGURE 6:**
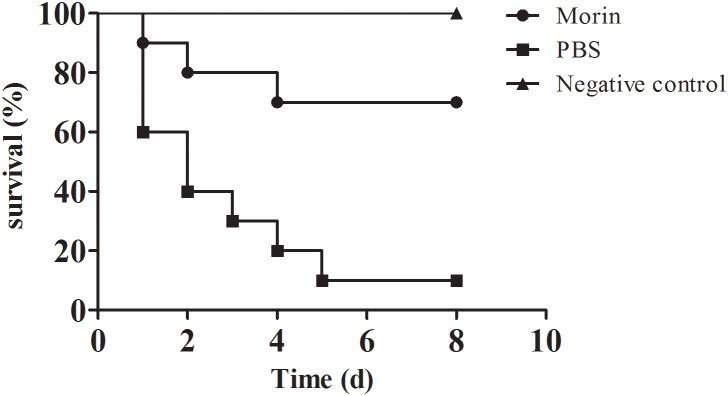
Morin treatment reduce the mortality of channel catfish infected with *A. hydrophila*. Infected channel catfish were administered with 25 mg/kg morin or PBS as positive control, the mortality of channel catfish was monitored for 8 days. The mortality for morin-treated group is significantly different from group for positive control when analyzed by log-rank test (*p* = 0.026 for morin-treated group).

According to previous studies, the pathogenicity of *A. hydrophila* strains lacking of *aerA* gene was significantly reduced, which revealed that AerA played an important role in *A. hydrophila* infections (Chakraborty et al., 1987). Our results have shown that morin could significantly reduce the hemolytic activity of AerA and protect Vero cells from cell injury mediated by AerA *in vitro*, which indicated that morin had potent protective effect against infections caused by *A. hydrophila in vivo*. Thus, an infection model of channel catfish was established to investigate the *in vivo* therapeutic effect of morin. Channel catfish infected with *A. hydrophila* (3 × 10^7^ CFU per fish) were then treated with either 25 mg/kg morin or sterilized PBS as control. As shown in Figure 6, deaths were observed 24 h postinfection. Skin of fish infected with *A. hydrophila* alone exhibited swelling and ulcers. Fish administered with PBS resulted in 90% death, while 30% of fish treated with 25 mg/kg morin (Figure 6). In conclusion, morin could significantly reduce the mortality of channel catfish infected with *A. hydrophila* (*p* = 0.026).

## Discussion

Large amount of antibiotics were discovered and used for the treatment of human infectious diseases, as well as for animal husbandry and aquaculture since the discovery of penicillin (Nikaido, 2009). Though the discovery and application of antibiotics reduced the mortality of infectious diseases to some extent, the abuse of antibiotics brought up new challenges. Following the stress of antibiotics on the life of bacterial pathogens, drug resistant, even multi-resistant strains were emerged. The spread of antibiotic resistance resulted in failure of antibiotic treatment. *A. hydrophila*, a typical pathogenic strain of aquatic environment, has been recognized as a human pathogen responsible for several diseases in human and other terrestrial animal worldwide (Stratev and Odeyemi, 2016). Because of the rapid development of aquaculture and the widespread use of antibiotics, multi-resistant *A. hydrophila* strains were isolated to be resistant to several antibiotics, including quinolones and tetracycline which are frequently used in aquaculture (Deng et al., 2014). The increasing incidence of *A. hydrophila* resistance has become a new public health concern. Thus, novel strategies to control bacterial infections caused by resistant *A. hydrophila* are urgently needed (Defoirdt et al., 2011). Anti-virulence therapy, targeting on virulence expression, activity, or regulation rather than inhibiting the growth of bacteria, has been introduced and was well investigated (Clatworthy et al., 2007). Pore-forming toxins (PFTs) are one of the most common bacterial extracellular proteins and are critical for the pathogenicity in a mount of bacteria, such as *Staphylococcus aureus* (*S. aureus*), *Streptococcus pneumoniae* (*S. pneumoniae*), *A. hydrophila*, *Clostridium perfringens* (*C. perfringens*), and *Escherichia coli* (*E. coli*) (Los et al., 2013). PFTs have been identified as unique targets for novel drugs against these pathogens because of their universal presence (Los et al., 2013). Studies using PFTs as target have shown significant effect against bacterial infections both *in vitro* and *in vivo* (Dong et al., 2013; Li et al., 2015; Song et al., 2016).

As is known, the pathogenesis of *A. hydrophila* was contributed by expression and secretion of extracellular virulence factors (Singh et al., 2013). Among them, two different kinds of PFTs named AerA and HlyA play important roles in infections caused by *A. hydrophila*. According to previous studies, the hemolytic activity was eliminated only when both HlyA (*hlyA*) and AerA (*aerA*) gene were double mutated (Wong et al., 1998). Moreover, the *hlyA* and *aerA* gene were identified in all the virulent *A. hydrophila* strains (Heuzenroeder et al., 1999). Consequently, AerA and HlyA were recognized as potent targets in identifying novel drugs (Dong et al., 2017a). Several works have determined the structure of AerA and mechanism of forming pore (Parker et al., 1994; Degiacomi et al., 2013). AerA is a member of toxins containing a high percentage of β-sheet. Following binding on the membranes of target cells, the heptamer with a 2 nm channel pore was formed which resulted in ion fluxes leading to cell death (Abrami et al., 2000; Bischofberger et al., 2016). Moreover, previous studies have shown that the pathogenesis of *A. hydrophila* strain mutated *aerA* to a mouse model was significantly decreased compared with the WT strain (Chakraborty et al., 1987). All these findings revealed that AerA could be chosen as a convincing target in drug discovery.

There have been several successful attempts identifying small molecules inhibiting the activity or expression of AerA and antibodies. In our previous study, we found that magnolol isolated from traditional Chinese medicine could significantly reduce the mortality of channel catfish infected with *A. hydrophila* via inhibiting the transcription of *aerA* (Dong et al., 2017a). In this paper, another natural compound named morin was identified that could directly inhibit the activity of AerA. Morin has been reported that could reduce the mortality of mice from pneumonia caused by *S. aureus* by blocking the activity of α-toxin produced by *S. aureus* (Wang et al., 2015). Similar results were has been identified that morin could reduce the *Streptococcus suis* pathogenicity in a mice model by neutralizing the activity of suilysin (Li et al., 2017). However, the effect of morin on *A. hydrophila* is not investigated. Compared with four kinds of indolo[3,2-*b*] quinoline, compounds against the activity of aerolysin-like hemolysin (ALH) produced by *A. sobria*, the mechanism and binding site of morin inhibiting AerA were determined using molecular dynamics and fluorescence quenching methods (Takahashi et al., 2016). Moreover, the *in vivo* effect against *A. hydrophila* infection has been evaluated by a channel catfish model. As expected, 25 mg/kg morin could provide a protection of 70% to fish infected with *A. hydrophila*. Rosmarinic acid has been reported that could inhibit the hemolysis induced by *A. hydrophila* supernatant by down-regulate the transcription of *aerA* and *ahh1* (Rama Devi et al., 2016). The inhibiting dose of rosmarinic acid was much higher than morin in this report, but several factors regulated by the quorum sensing system was suppressed and similar *in vivo* effect was achieved (Rama Devi et al., 2016). Some metal ions such as Zinc, cupric, and cadmium have been identified as inhibitors of hemolysis caused by AerA. However, the inhibition only occurred when continuous ions were presented, which limited the application under field conditions (Avigad and Bernheimer, 1976). Another approach against AerA is vaccine. Several different types of vaccines have been identified in recent years. Although some of them showed higher protective effects against *A. hydrophila* infections than small molecules, the protection against different *A. hydrophila* isolates needs to be further studied (Anuradha et al., 2010). Although these findings have shown a convincing results against *A. hydrophila* both *in vitro* and *in vivo*, more efforts are still needed before morin can be used in aquaculture farming and human clinic. Moreover, the risk of *A. hydrophila* infections after antibiotic treatment has not been clarified, thus antibiotic treatment combined with morin is not recommended before any experimental data was carried out. Despite this, the findings provided a new approach in identifying novel drugs against *A. hydrophila* infections. Collectively, it is reasonable to infer that morin may be a potential agent for the treatment of infections caused by resistant *A. hydrophila*.

## Author Contributions

JD and XA conceived the project. JD, XA, and YL designed the experiments. YL, NX, and QY performed the experiments. JD and XA wrote the paper and all authors made the editorial input.

## Conflict of Interest Statement

The authors declare that the research was conducted in the absence of any commercial or financial relationships that could be construed as a potential conflict of interest.

## Footnotes

1 http://www.rcsb.org/pdb/home/home.do
