# Supplementary material for: Morin Protects Channel Catfish From Aeromonas hydrophila Infection by Blocking Aerolysin Activity
Source: Front Microbiol. 2018 Nov 21;9:2828. doi: 10.3389/fmicb.2018.02828 (PMC6258893; doi:10.3389/fmicb.2018.02828)
Supplement: Supplementary file 1 [file Data_Sheet_1.PDF]

Supplementary Material

Morin protects channel catfish from *Aeromonas hydrophila* infection by blocking aerolysin activity

Jing Dong [1, 2], Yongtao Liu [1, 2], Ning Xu [1, 2], Qiuhong Yang [1, 2], Xiaohui Ai [1, 2*]

1 Yangtze River Fisheries Research Institute, Chinese Academy of Fishery Sciences, Wuhan 430223, China;

2 Key Laboratory of Control of Quality and Safety for Aquatic Products, Ministry of Agriculture, Beijing 100071, China.

*** Correspondence:** Corresponding Author: aixh@yfi.ac.cn

1 Supplementary Figures and Tables

1.1 Supplementary Figures

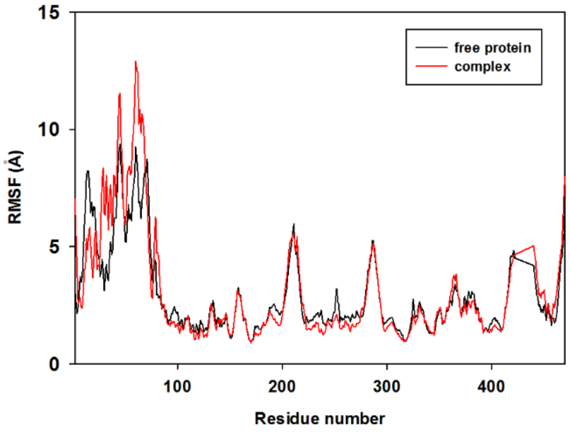

Supplementary Figure 1. The root-mean-square deviations (RMSDs) of all the atoms of pAerA-morin complex and free pAerA with respect to its initial structure as function of time.

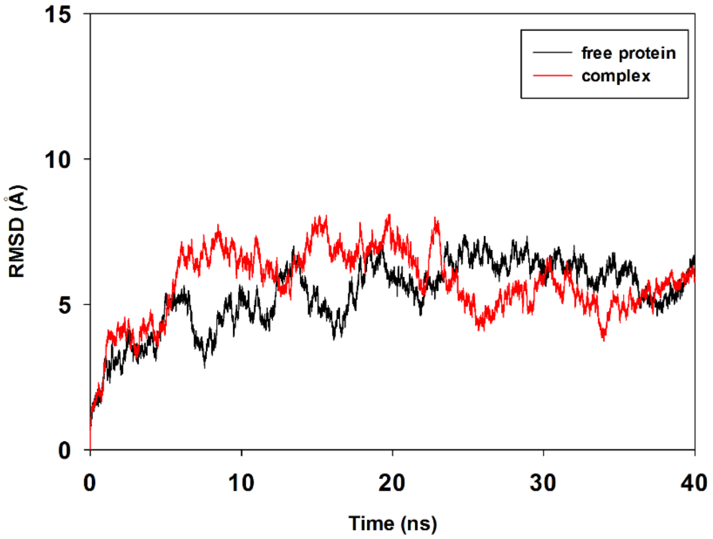

Supplementary Figure 2. RMSF of the residues of the whole protein in pAerA-morin complex and free pAerA during the 40 ns simulation.

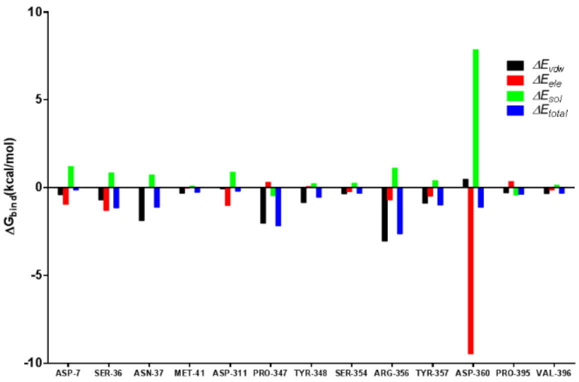

Supplementary Figure 3. Decomposition of the binding energy on a per-residue basis in the pAerA-morin complex.

1.2 Supplementary Tables

Supplementary Table 1 Primer pairs used for the mutants

Primer Name	Sequence
R356A-F	5’-GGCGAGCAGCATTGCATACCAGTGGGAC-3’
R356A-R	5’-GTCCCACTGGTATGCAATGCTGCTCGCC-3’
P347A-F	5’-CCTTCGTCATCGGGGCGTACAAGGACAAG-3’
P347A-R	5’-CTTGTCCTTGTACGCCCCGATGACGAAGG-3’
S36A-F	5’-GGAAGCCCAGAGCGTTAAAGCGAATATTGTCAATATGATGG-3’
S36A-R	5’-CCATCATATTGACAATATTCGCTTTAACGCTCTGGGCTTCC-3’
